# Inhibition of Hsp90 K284 Acetylation Aalleviates Cardiac Injury After Ischemia–Reperfusion Injury

**DOI:** 10.1007/s12265-024-10548-0

**Published:** 2024-07-24

**Authors:** Dongyu Zhan, Na Zhang, Li Zhao, Zhirui Sun, Chunyang Cang

**Affiliations:** Department of Cardiovascular Medicine, The Third Affiliated Hospital of Qiqihar Medical University, No. 3, Taishun Street, Tiefeng District, Qiqihar, 161099 Heilongjiang Province P. R. China

**Keywords:** Hsp90, NF-κB p65, Acetylation, Ischemia‒Reperfusion Injury, IL-37

## Abstract

Our objective was to determine the role of acetyl-Hsp90 and its relationship with the NF-κB p65 signaling pathway in CVDs. We investigated the effect of acetyl-Hsp90 on cardiac inflammation and apoptosis after ischemia–reperfusion injury (I/RI). The results showed that the induction of acetyl-Hsp90 occurred in the heart during I/R and in primary cardiomyocytes during oxygen–glucose deprivation/reoxygenation (OGD/R). Moreover, the nonacetylated mutant of Hsp90 (Hsp90-K284R), through the regulation of ATPase activities within its N-terminal domain (NTD), indirectly or directly increases its interaction with NF-κB p65. This led to a reduction in the activation of the NF-κB p65 pathway, thereby attenuating inflammation, apoptosis, and fibrosis, ultimately leading to an improvement in cardiac function. Furthermore, we demonstrated that recombinant human interleukin-37 (rIL-37) exerts a similar cardioprotective effect by reducing acetylation at K284 of Hsp90 after inhibiting the expression of KAT2A.

**Graphic Abstract:**

Hsp90 is acetylated by KAT and can be deacetylated by KDAC, which is in balance in the steady state. Moreover, Hsp90 interacts with NF-κB p65 in the cytosol and inhibits p65 translocation into the nucleus. However, Hsp90-K284 can be acetylated by KAT2A after ischemia–reperfusion treatment. Subsequently, the protein–protein interaction between Hsp90 and NF-κB p65 was disturbed, which induced NF-κB p65 to translocate into the nucleus. However, rIL-37 disturbs this phenotype by inhibiting KAT2A activity.

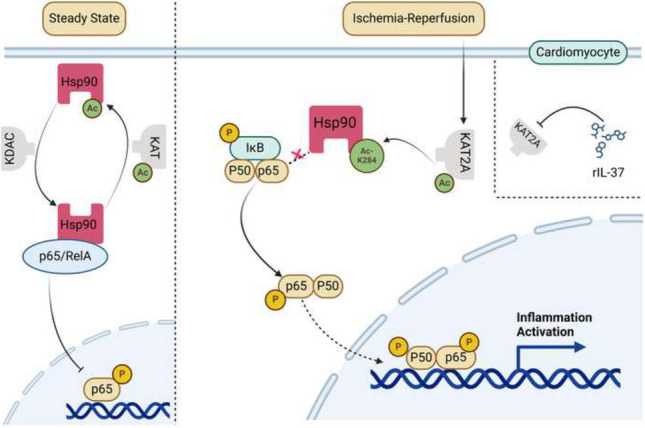

**Supplementary Information:**

The online version contains supplementary material available at 10.1007/s12265-024-10548-0.

## Introduction

The incidence of cardiovascular diseases (CVDs), especially ischemic cardiomyopathy, remains high worldwide [1]. The conventional approach to rescuing ischemic cardiomyocytes involves re-establishing blood flow through recanalization using thrombolysis, percutaneous coronary intervention, or surgical approaches [2,3]. However, researchers found that the blood supply of ischemic tissue is restored, but the damage to cell function will be aggravated, which is called ischemia–reperfusion injury (I/RI) [4]. Myocardial I/RI further induces pathological and physiological changes and microenvironment changes in the heart, especially the activation of inflammatory cells and apoptosis of cardiomyocytes [5], which lead to further cardiac dysfunction in patients. Therefore, I/R injury brings challenges to reperfusion therapy for ischemic diseases represented by acute myocardial infarction (AMI), which prompts scientists to conduct in-depth research on the underlying molecular mechanism of myocardial reperfusion injury. Developing new therapeutic approaches to improve cardiomyocyte survival has significant clinical implications [4].

I/RI is an extremely complex process involving the precise expression of many genes in the process of injury and repair [6]. Furthermore, posttranslational modifications of proteins also play a powerful role in CVDs, such as phosphorylation [7], methylation [8], and acetylation [9]. Under normal conditions, the acetylations mediated by lysine acetyltransferases (KATs) and lysine deacetylases (KDACs) tend to be stable. However, following stimulation, the homeostasis of acetylations mediated by KAT and KDAC is disrupted. Wang found that increased acetyl-Drp1 can induce cardiomyocyte death and cardiac dysfunction [10]. In addition, Vernon W Dolinsky also found that SIRT3 can prevent doxorubicin-induced dilated cardiomyopathy by regulating protein acetylation [11].

Considering the significant role of posttranslational modifications, exploring the acetylations of key genes in CVDs has emerged as a central focus of research. The heat shock protein (Hsp) family is a highly conserved protein family, which is a molecular chaperone necessary to maintain the stability and normal function of intracellular proteins and is also recognized as a protective molecule closely related to stress tolerance [12]. Several studies have highlighted the role of Hsp90 in stress-induced inflammation and apoptosis by recruiting its interacting molecules in CVDs [13]. In addition, Hsp90 is regulated by posttranslational modifications, such as acetylation [14]. The acetylation of Hsp90 has been reported to be involved in endothelial dysfunction in liver injury [15] and cancers [16]. However, studies on the acetylation of Hsp90 in the context of I/R remain limited.

Interleukin-37 (IL‐37) is mainly expressed in peripheral blood mononuclear cells and acts as a natural suppressor of innate and adaptive immune response [17]. IL-37 can suppress inflammasome activation and inflammatory cytokines production by translocating to the nucleus [18]. The role of IL-37 in cardiovascular disease has attracted researchers' attention, and its protective effect on CVDs has been widely recognized [19–21]. MI patients undergoing coronary recanalization show increased plasma IL-37 [22]. In addition, reduced IL-37 expression may cause inflammation and increase the risk of coronary artery disease [23]. Currently, one primary mechanism through which IL-37 operates relies on the IL-37 cell surface receptor (IL-18R1) [24]. Additionally, it has been reported that Stat4 binds to Il-18R1 and transiently regulates acetylated histones H3 and H4 [25]. However, it remains unknown whether IL-37 regulates acetylation in the process of MI and, if so, what mechanisms are involved.

Thus, in this study, we aimed to determine the role of acetylated Hsp90 with rIL-37 in I/RI. We believe that exploring the regulatory mechanism of Hsp90 acetylation may become a new therapeutic target.

## Methods

### Animal Model and rIL-37 Administration

The ten-week-old C57BL/6 male mice (22 ~ 25 g) involved in this study were purchased from Charles River Company, China, and mice were randomly assigned to each group, n = 6. In addition, all in vivo experiments and operations strictly followed the Guide for the Care and Use of Laboratory Animals, which was published by the U.S. National Institutes of Health. Isoflurane was used to anesthetize the mice, and then the trachea was connected to a ventilator. The skin was cut along the fourth rib on the left side of the sternum to expose the chest cavity. The left coronary artery was exposed after cutting the pericardium, and an 8–0 thread was used to ligate the left coronary artery to a depth of approximately 1 mm. Next, the PE10 tube was passed through the ligature to compress the coronary artery. At the same time, mice were injected once in the area of left ventricles with 20 μL containing 5 × 10^10^ genome copies of recombinant adeno-associated virus 9 (rAAV9), which drives the expression of wild-type Hsp90 (Hsp90-WT) or the Lys284-acetylation site mutation Hsp90-K284R, was constructed by Shanghai Heyuan Biotechnology Company (Shanghai, China). After 1 h of ischemia, the PE10 tube was removed to cause myocardial reperfusion. A 5–0 thread was used to close the chest cavity, and the muscle and chest skin were sterilized by iodophor.

rIL-37 (Adipogen AG, Liestal, AG-40A-0174Y-3010, Switzerland) was dissolved in phosphate-buffered saline (PBS, P4417, Merck, Germany) and stored at -20 °C. rIL-37 was administered to mice *i.v.* at a 1 μg/day dose, twice per week after I/R surgery.

## Echocardiography

Mouse cardiac function was detected by the Vevo 770 UBM system (VisualSonics Inc., Toronto, ON, Canada) 28 days post I/R surgery. After anesthetization, the mice were immobilized on the detector in a supine position. By continuously monitoring the left ventricular end-diastolic diameter (LVEDD) and left ventricular end-systolic diameter (LVESD) data during three cardiac cycles, the left ventricular ejection fraction (EF%) and left ventricular fractional shortening (FS%) were subsequently calculated according to the echocardiography software. The experiment was strictly according to the double-blind method.

## Histology

Mouse heart tissues were harvested after anesthetization with medetomidine-midazolam-fentanyl. The chest was opened with surgical scissors along the thoracic spine. Then, the heart was placed in PBS to remove residual blood in the chamber. Tissues were fixed in 4% paraformaldehyde (PFA) solution for 2 h and then infiltrated overnight in 30% sucrose solution. For tissue-frozen sections, the optimal cutting temperature compound (OCT compound) was used for tissue embedding, and then the tissues were cut into 5 μm sections and placed at -80 °C for long-term storage. According to the standard protocol of the Picrosirius Red staining kit (Abcam, ab150681, England), the heart tissues were incubated with Picrosirius Red reagent for 1 h. The fields of view were observed and imaged under an optical microscope (Carl Zeiss, Thuringia, Germany). ImageJ software was used to analyze the area of collagen.

## Primary Cell Extraction

The primary cardiomyocytes involved in this study were extracted from the hearts of C57BL/6 mice (2 to 3 days after birth). The hearts of neonatal mice were sectioned first, and then the tissues were digested with a solution containing 0.25% trypsin and 0.1% collagenase II. The separated cells were incubated in the plate for 1.5–2 h. Afterward, the cardiomyocytes were separated from the fibroblasts using differential adhesion. Purified primary cardiomyocytes were cultured in Dulbecco's modified Eagle's medium (DMEM, Thermo Fisher, 11965092, USA) containing 10% horse serum and 5% fetal bovine serum (FBS, Thermo Fisher, A5256801, USA) in a 37 °C incubator with 95% O_2_ and 5% CO_2_.

## Cell Culture and Treatment

After 1 day of preculture, primary cardiomyocytes overexpressing Hsp90-WT or Hsp90-K284R (Ad5-HA-Hsp90-WT and Ad5-HA-Hsp90-K284R, respectively, which were constructed by KeyGen Biotech) were transfected with Lipofectamine^3000^ according to the standard protocol. In addition, cardiomyocytes were challenged with 20 ng/mL rIL-37 for 3 h. Then, cardiomyocytes were cultured with oxygen–glucose deprivation (OGD) conditions (deoxygenated, no glucose, DMEM) for 2 h at 37 °C in a hypoxic chamber containing 95% N_2_ and 5% CO_2_. Subsequently, cardiomyocytes were returned to a 37 °C incubator with 95% O_2_ and 5% CO_2,_ provided with fresh complete medium, and incubated for an additional 1 h under oxygen–glucose deprivation/reoxygenation (OGD/R) conditions.

## Immunofluorescence Staining

Before staining, 4% PFA was used to fix heart tissues or primary cardiomyocytes. After washing with PBS, the cardiomyocytes or heart tissues were permeabilized with Triton X-100 (Carl Roth GmbH, 3051.2, Germany) for 15 min, followed by incubation with 10% goat serum for half an hour. Subsequently, the primary antibodies of anti-cTnI (Invitrogen, 701,585, USA) at 1:1000, anti-NF-κB p65 (Invitrogen, 436,700, USA) at 1:500, anti-α-SMA (Thermo Fisher, 55,135–1-AP, USA) at 1:500, anti-CD68 (Abcam, ab283654, England) at 1:500, and anti-Hsp90 (Abcam, ab59459, England) at 1:300 dilution in PBS containing 5% BSA and 0.2% Tween®20 were used to incubate the samples at 4 °C overnight. After washing with PBS, the conjugated secondary antibodies (AlexaFluor®488, 1:1000, Invitrogen, A33077, USA; AlexaFluor®555, 1:1000, Invitrogen, A20009, USA) were used to conjugate the primary antibody in the dark for 1 h. Subsequently, DAPI (1:1000, Sigma Aldrich, 10,236,276,001, USA) was diluted at 1:1000 in PBS for nuclear staining in the dark for 10 min. The Wheat Germ Agglutinin (WGA, 1:100, Thermo Fisher, W7024, USA) staining kit was used according to the standard protocol to detect the degree of cardiac fibrosis. According to the standard protocol, a TUNEL staining kit (KeyGen Biotech, KGA700, China) was used to detect cardiomyocyte apoptosis. After incubation with the reaction buffers, the fields of view were observed and taken under a fluorescence microscope (Carl Zeiss, Thuringia, Germany). ImageJ software was used to analyze the proportion of positive cells.

## Western Blotting

The total protein of cells or tissues was extracted by NP-40 lysis buffer. The boiled protein samples were separated on a polyacrylamide gel by protein gel electrophoresis and transferred from the gel to a polyvinylidene fluoride (PVDF) membrane. After blocking the membrane with 5% milk buffer, the membrane was incubated with primary antibodies (Hsp90, 1:2000, Abcam, ab59459, England; acetyl-Hsp90K284, 1:500, antibodies-online, ABIN3181886, Germany; NF-κB p65, 1:2000, Invitrogen, 436700, USA; phospho-NF-κB p65 (Ser536), 1:3000, Invitrogen, 82335–1-RR, USA; Bax, 1:3000, Abcam, ab32503, England; Bcl-2, 1:3000, Abcam, ab182858, England; KAT2A/GCN5, 1:2000, Abcam, EPR21146, England; β-actin, 1:5000, CST, #4970, United State; Lamin A, 1:2000, CST, #4777, United State) at 4 °C overnight. After washing with PBST, the membrane was incubated with HRP-labeled secondary antibody (mouse, 1:10000, CST, 7076, rat, 1:5000, Abcam, ab6734; rabbit, 1:5000, Abcam, ab6728) at room temperature for 1 h. Super sensitivity substrate (Thermo Fisher, A38556, USA) was subsequently used to develop the membrane. For the coimmunoprecipitation (Co-IP) assay. The primary antibodies HA (Invitrogen, 26183, USA), acetyl-lysine (CST, 9441, USA), and control IgG (CST, 4410, USA) were incubated with the agarose beads first, and then the beads were incubated with total proteins to pull down the interacting proteins. In addition, the nuclear protein of cardiomyocytes was extracted by using fractionation buffer (Abcam, ab109719, England). For quantification, Image Lab software was used to analyze the protein expression according to the host-keeping gene.

## RNA Isolation and Quantitative Reverse-Transcription Polymerase Chain Reaction (qRT‒PCR)

Total RNA from tissues or cardiomyocytes was isolated according to the standard protocol of the ISOLATE II RNA Mini Kit (Bioline, BIO-52073, England). Subsequently, qualified RNA was reverse transcribed into cDNA by a PrimeScript RT Master Mix kit (TaKaRa, RR036A, Japan). AceQ qPCR SYBR Green (Vazyme, Q111-02, China) was used for quantification for cDNA amplification. The mRNA expression level of the target gene was determined according to the formula: Relative gene expression = 2^−ΔΔCt^. GAPDH was used to normalize the expression of mRNA. Hsp90 primer: forward 5'- > 3: AATTGCCCAGTTAATGTCCTTGA; reverse 5'- > 3: CGTCCGATGAATTGGAGATGAG. GAPDH primer: forward 5'- > 3: AGGTCGGTGTGAACGGATTTG; reverse 5'- > 3: TGTAGACCATGTAGTTGAGGTCA.

## Enzyme-Linked Immunosorbent Assay (ELISA)

The serum of mice or the supernatant of primary cardiomyocytes was harvested after centrifugation to detect the expression of inflammatory cytokines. The samples were processed according to the standard protocol of the Invitrogen ELISA kit (IL-1β, BMS6002; IL-6, BMS603-2; TNF-α, BMS607-3). After the addition of the stop solution, a microplate reader was used to record the OD values at 450 nm. According to the standard curve, the protein expression of inflammatory cytokines was calculated by Origin software.

## ATPase Activity Assay

Cold ATPase assay buffer was used to lyse the cells, and the supernatant was subsequently harvested. Samples were reacted with Reaction Mix (ATPase Assay kit, Abcam, ab234055, England) at 25 °C for 30 min. Then, 30 µL ATPase Assay Developer was added to the reactions and incubated at 25 °C for 30 min. The OD value at 650 nm was detected by a microplate reader, and the ATPase activity was calculated according to the standard formula.

## Cell Apoptosis and Viability Assay

Primary cardiomyocytes were collected after transfection or drug challenge with or without OGD/R treatment. To detect the apoptosis of cardiomyocytes, cardiomyocytes were incubated with Annexin V-fluorescein isothiocyanate (V-FITC) and propidium iodide (PI) according to the apoptosis kit (KeyGen, RCFK302, China), and then the apoptosis rate of cardiomyocytes was detected by FACS (Thermo Fisher, USA). Moreover, primary cardiomyocytes from 96-well plates were used to detect cell viability by using a cell counting kit 8 (CCK8, Abcam, ab228554, England). After incubation with CCK8 buffer for 2 h, the OD values were recorded with a microplate reader at 450 nm. The data were analyzed according to the formula: Cell viability rate = [(As-Ab)/(Ac-Ab)] × 100%.

## Molecular Docking

Molecular docking was performed using AutoDock Vina 1.1.2 software. The 3D structure of the protein used for docking was derived from UniProt: Hsp90 (ID: Q7L3B6) and NF-kB (ID: Q04206). Visual analysis of docking results using PyMol software.

## Statistical Analysis

Data are expressed as the mean ± standard error of the mean (S.E.M). All statistical data were analyzed using GraphPad Prism 8.0. For the comparison of means between the two groups, an unpaired t test was performed when the data were normally distributed; otherwise, a Welch t test was performed. A one-way analysis of variance (Tukey's multiple comparison test) was used to determine significant differences for comparisons between multiple groups. Differences were considered statistically significant when P ≤ 0.05.

## Results

### Hsp90 Acetylation was Driven by I/R in Mice and OGD/R in Primary Cardiomyocytes

**Fig. 1:**
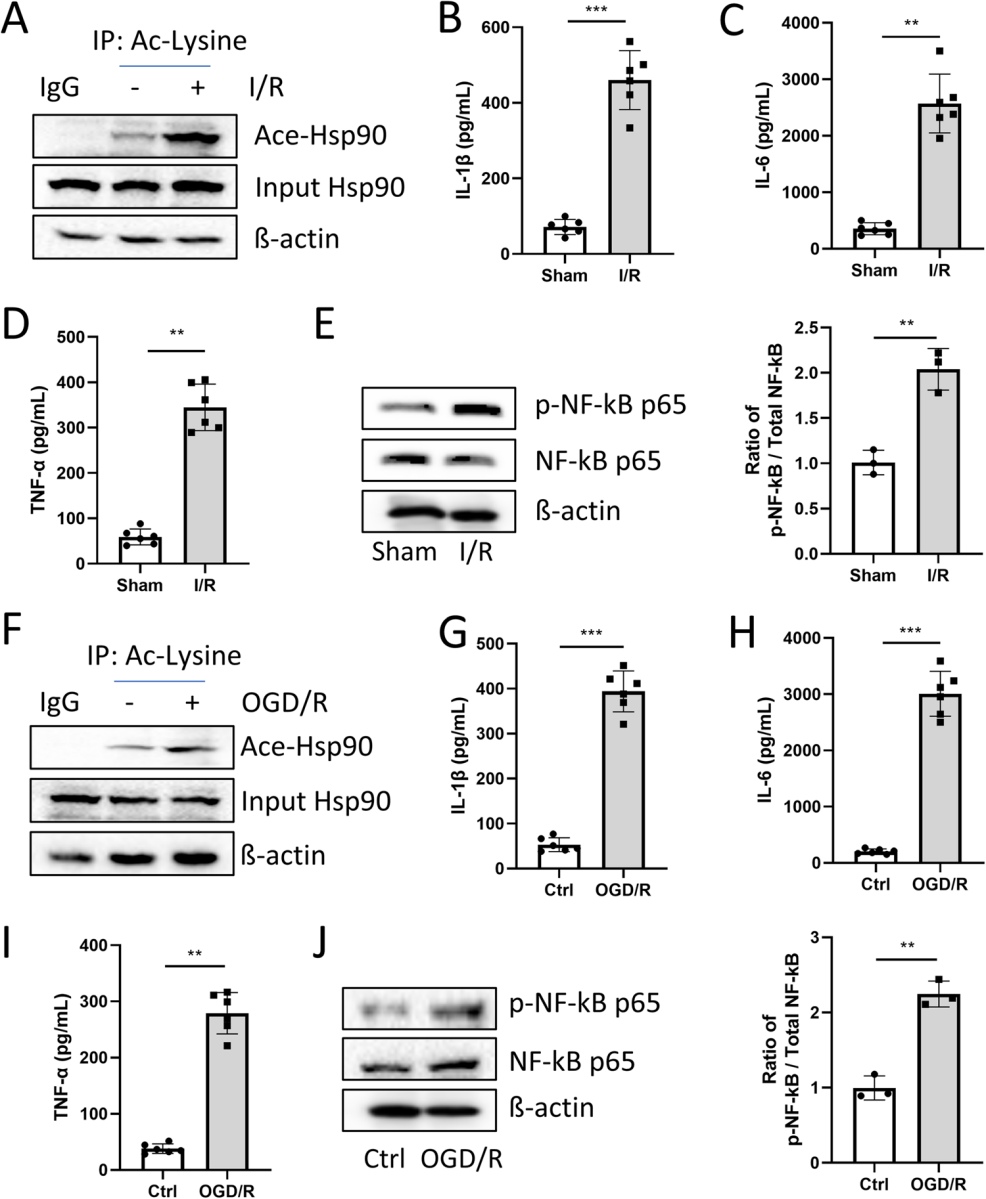
Hsp90 acetylation was driven by ischemia–reperfusion in mice and OGD/R in primary cardiomyocytes. (**a**)**.** Acetylated Hsp90 and total Hsp90 protein expression were detected by Co-IP assay. (**b-d**)**.** Inflammatory cytokines: IL-1β, IL-6, and TNF-α protein levels were detected by ELISA. n = 6 for each group. (**e**)**.** Phosphorylated NF-κB p65 and total NF-κB p65 protein expression were detected by Western blotting. n = 3 for each group. Myocardial infarction (MI)tissues and serum were taken 3 days or 28 days post-I/R surgery, respectively. **p < 0.01, ***p < 0.001 *vs.* Sham. (**f**)**.** Acetylated Hsp90 and total Hsp90 protein expression were detected by Co-IP assay. (**g-i**)**.** Inflammatory cytokines: IL-1β, IL-6, and TNF-α protein levels were detected by ELISA. n = 6 for each group. (**j**)**.** Phosphorylated NF-κB p65 and total NF-κB p65 protein expression were detected by Western blotting. n = 3 for each group. Cardiomyocytes or supernatants were harvested 1–2 days post-OGD/R challenge. Primary cardiomyocytes and supernatants were harvested post-OGD/R treatment. Image Lab was used to analyze the protein expression compared with the host-keeping gene β-actin. **p < 0.01, ***p < 0.001 *vs.* Ctrl

We observed that acetyl-Hsp90 protein levels increased by more than threefold, while the expression of Hsp90 remained unchanged in mouse hearts in I/R (Fig. 1a). At the same time, I/R increased the levels of inflammatory markers in mouse plasma (Fig. 1b-d). Moreover, an elevated ratio of phosphorylated NF-κB p65/total NF-κB p65 (p-p65/p65) was observed in mouse hearts after I/R (Fig. 1e). In neonatal mouse primary cardiomyocytes subjected to OGD/R, a notable increase of acetyl-Hsp90 level was found (Fig. 1f). Furthermore, OGD/R treatment led to upregulated inflammatory marker levels in the cell supernatant and an increased ratio of p-p65/p65 in primary cardiomyocytes (Fig. 1g-j). To explore the function of acetyl-Hsp90 in cardiomyocytes, we utilized the CSS-Palm website (http://csspalm.biocuckoo.org/) for prediction of acetylation sites of Hsp90. The 284 K site of Hsp90 was predicted to be acetylated. Additionally, according to a previous paper^15^, arginine (R) mutations cannot be acetylated by acetylation enzymes. Consequently, we generated a mutant form of Hsp90 at site 284 (Hsp90-K284R) to explore its functional implications. (Supplementary Fig. 1).

### Inhibition of Hsp90 Acetylation Improved Mouse Cardiac Function after I/R

**Fig. 2:**
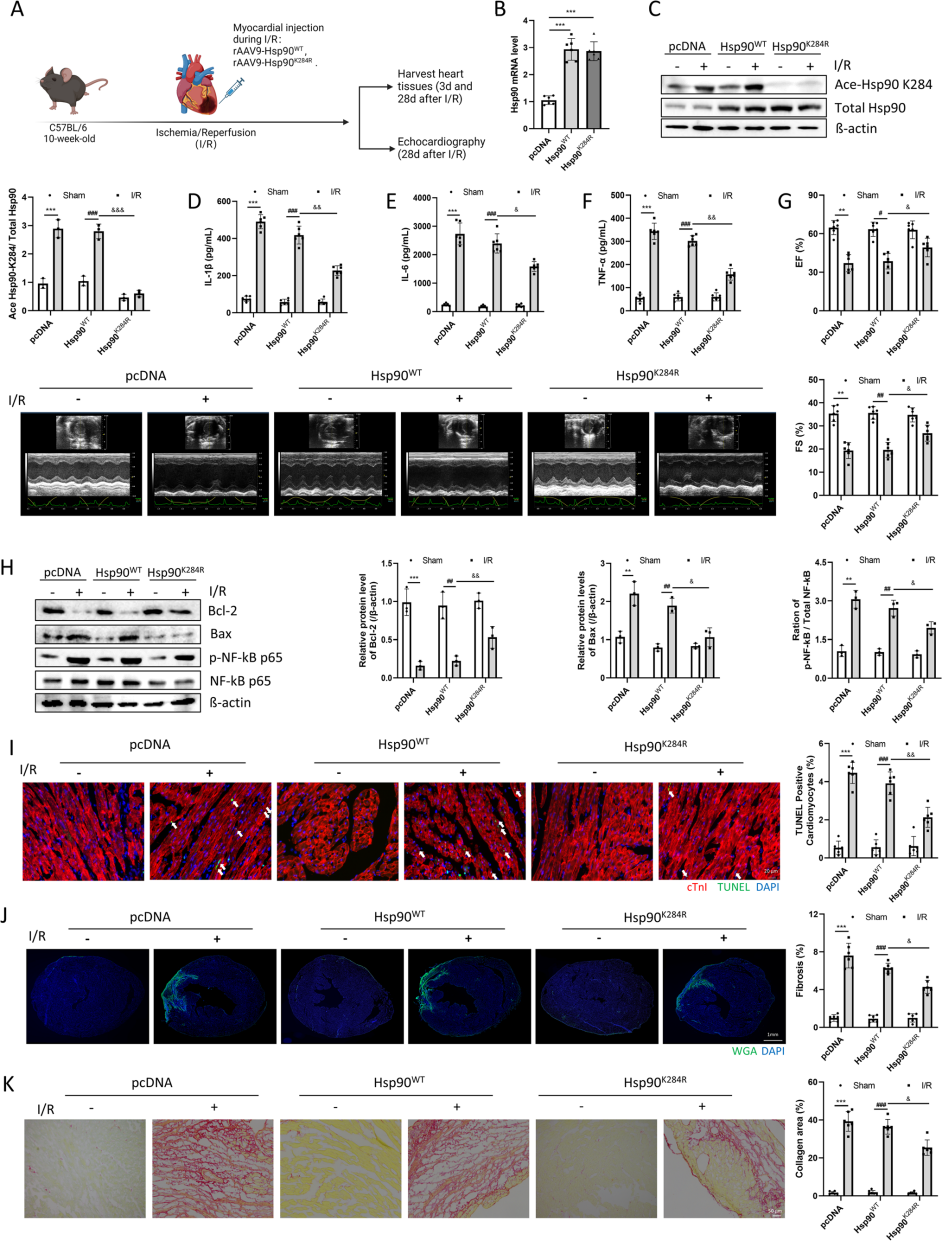
Inhibition of Hsp90 acetylation improved cardiac function after I/R in mice.(**a**). Study design of ischemia/reperfusion experiments in C57BL/6 mice. Mice were challenged with rAAV9-Hsp90-WT and rAAV-Hsp90-K284R during I/R surgery. (**b**)**.** Hsp90 mRNA levels in mouse hearts after rAAV treatment were detected by qPCR. n = 6 for each group. ***p < 0.001 *vs.* pcDNA. (**c**)**.** Ace-Hsp90 K284 and total Hsp90 protein expression were detected by Western blotting. n = 3 for each group. (**d-f**)**.** Inflammatory cytokines: IL-1β, IL-6, and TNF-α protein levels were detected by ELISA. n = 6 for each group. (**g**)**.** Cardiac function was detected by echocardiography (LVEF% and LVFS%) 28 days post-I/R surgery. n = 6 for each group. (**h**)**.** Bcl-2, Bax, phosphorylated NF-κB p65, and total NF-κB p65 protein expression were detected by Western blotting. n = 3 for each group. MI tissues and serum were taken 3 days or 28 days post I/R surgery, respectively. (**i**)**.** Myocardial apoptosis after 3 days of I/R surgery was detected by TUNEL staining (magnification: 400x; n = 6 for each group). (**j**)**.** Myocardial fibrosis was detected by WGA staining (Magnification: 100x; n = 6 for each group). (**k**)**.** Myocardial fibrosis was detected by picrosirius red staining (magnification: 200x; n = 6 for each group), and MI tissues were taken 28 days post-I/R surgery. ImageJ was used to analyze myocardial fibrosis. Image Lab was used to analyze the protein expression compared with the host-keeping gene β-actin. **p < 0.01, ***p < 0.001 *vs.* Sham + pcDNA. #*p* < 0.05, ##*p* < 0.01, ###*p* < 0.001 *vs.* Sham + pcDNA-Hsp90-WT. &*p* < 0.05, &&*p* < 0.01, &&&*p* < 0.001 *vs.* I/R + pcDNA-Hsp90-WT

The influence of Hsp90 acetylation on NF-κB p65 activity and inflammation remains uncertain. To explore the role of Hsp90 acetylation in I/R, Hsp90-WT or Hsp90-K284R rAAVs were injected into the endocardium during surgery (Fig. 2a). qPCR analysis revealed a significant increase in Hsp90 mRNA levels after rAAV injection compared with the pcDNA-Vector group (Fig. 2b). Likewise, the protein level of acetyl-Hsp90 K284 in the hearts of mice injected with rAAV9-Hsp90-K284R was significantly lower than that in the hearts of mice injected with Hsp90-WT (Fig. 2c). Additionally, mice in the Hsp90-K284R group exhibited reduced inflammation levels (Fig. 2d-f, Supplementary Fig. 2a). Echocardiographic results showed that rAAV9-Hsp90-K284R relieved EF% and FS% after I/R (Fig. 2g). In addition, mice after I/R injected with rAAV9-Hsp90-K284R had lower expression of Bax, higher expression of Bcl-2, and a lower ratio of p-p65/p65 compared with the Hsp90-WT group (Fig. 2h). The immunofluorescence staining results indicated fewer TUNEL-positive cardiomyocytes and less fibrosis in the rAAV9-Hsp90-K284R group than in the Hsp90-WT group (Fig. 2i and j). Moreover, picrosirius red staining also confirmed that the cardiac collagen area was significantly reduced in the Hsp90-K284R group compared with the Hsp90-WT group (Fig. 2k). However, myofibroblast density showed no significant difference between rAAV9-Hsp90-K284R group and the Hsp90-WT group (Supplementary Fig. 2c). In brief, these results indicated that inhibition of Hsp90 acetylation significantly decreased inflammatory levels, cardiac fibrosis and apoptosis, thereby improving cardiac function.

### Inhibition of Hsp90 Acetylation Decreased Inflammation and Apoptosis after OGD/R in Primary Cardiomyocytes

**Fig. 3:**
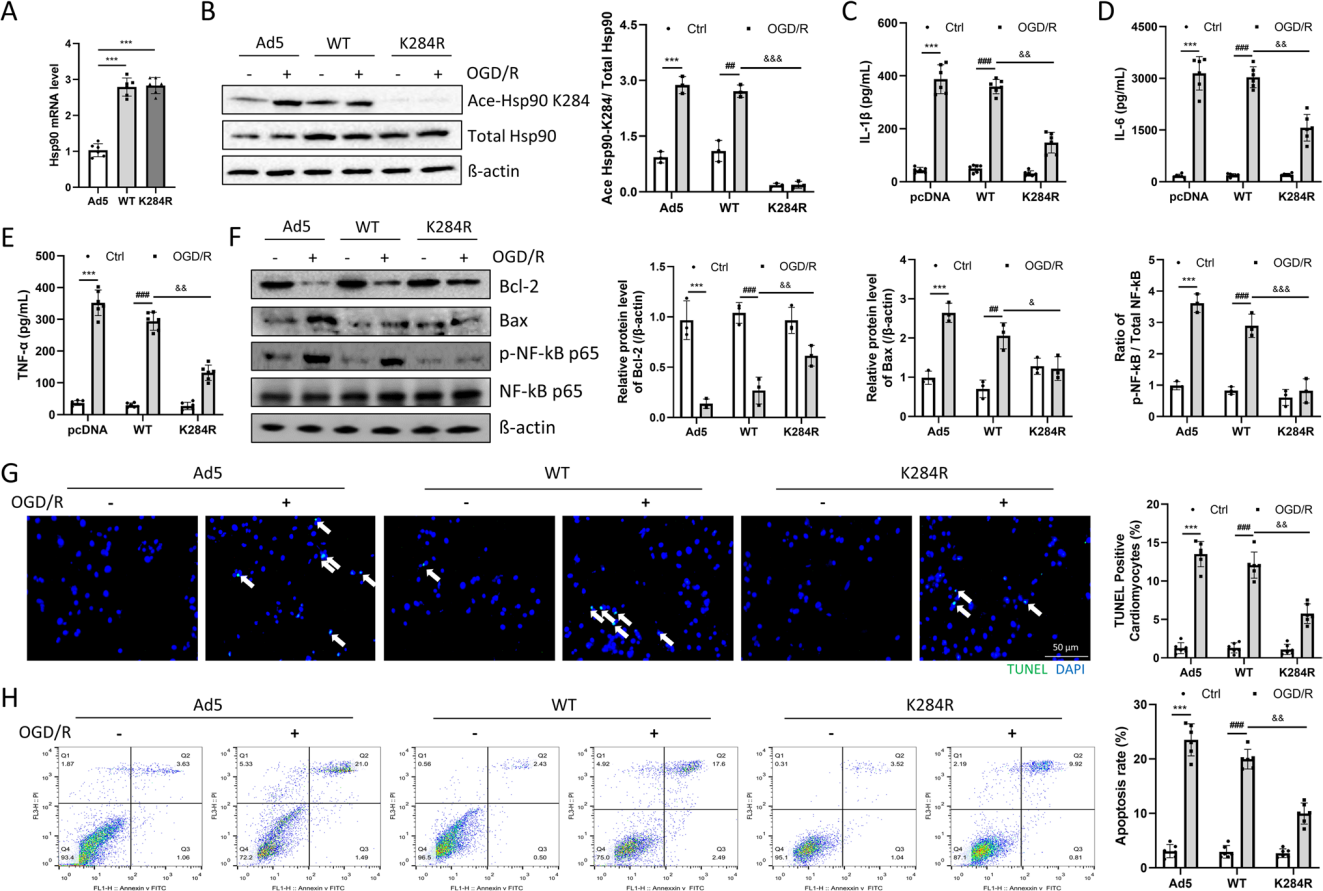
Inhibition of Hsp90 acetylation decreased inflammation and apoptosis after OGD/R in primary cardiomyocytes. (**a**)**.** Hsp90 mRNA levels in cardiomyocytes after adenovirus transfection were detected by qPCR. n = 6 for each group. ***p < 0.001 *vs.* Ad5-Vector. (**b**)**.** Ace-Hsp90 K284 and total Hsp90 protein expression were detected by Western blotting. n = 3 for each group. (**c-e**)**.** Inflammatory cytokines were detected by ELISA: IL-1β, IL-6, and TNF-α protein levels. n = 6 for each group. (**f**)**.** Bcl-2, Bax, phosphorylated NF-κB p65, and total NF-κB p65 protein expression were detected by Western blotting. n = 3 for each group. (**g** and **h**)**.** Cardiomyocyte apoptosis was detected by TUNEL staining and FACS (magnification: 200x; n = 6 for each group). Cardiomyocytes or supernatants were harvested 1–2 days post-OGD/R challenge. Image Lab was used to analyze the protein expression compared with the host-keeping gene β-actin. ***p < 0.001 *vs.* Ctrl + Ad5-Vector. ##*p* < 0.01, ###*p* < 0.001 *vs.* Crtl + Ad5-Hsp90-WT. &*p* < 0.05, &&*p* < 0.01, &&&*p* < 0.001 *vs.* OGD/R + Ad5-Hsp90-WT

To explore the role of acetyl-Hsp90 in vitro, primary cardiomyocytes from wild-type mice (2–3 days old) were isolated and then transfected with Ad5-Hsp90-WT or Ad5-Hsp90-K284R before OGD/R challenge. From the qPCR results, the mRNA level of Hsp90 was enhanced in the Ad5-Hsp90-WT or Ad5-Hsp90-K284R groups after transfection (Fig. 3a). The protein level of acetyl-Hsp90 K284 in the primary cardiomyocytes transfected with Ad5-Hsp90-K284R was significantly lower than that in the Ad5-Hsp90-WT group (Fig. 3b). Additionally, the protein levels of inflammatory cytokines were lower in the Ad5-Hsp90-K284R group (Fig. 3c-e). In addition, following OGD/R challenge of primary cardiomyocytes, overexpression of Hsp90-K284R led to increased Bcl-2 expression and decreased expression of Bax, and a reduced ratio of p-p65/p65 compared with the Hsp90-WT group (Fig. 3f). Furthermore, compared with the Ad5-Hsp90-WT group, overexpression of Hsp90-K284R reduced the apoptosis of cardiomyocytes induced by OGD/R (Fig. 3g and h). In summary, OGD/R-induced primary cardiomyocyte apoptosis and inflammation can be alleviated by inhibiting Hsp90 K284 acetylation.

### Hsp90 Acetylation Decreases its Interaction with NF-κB p65

**Fig. 4:**
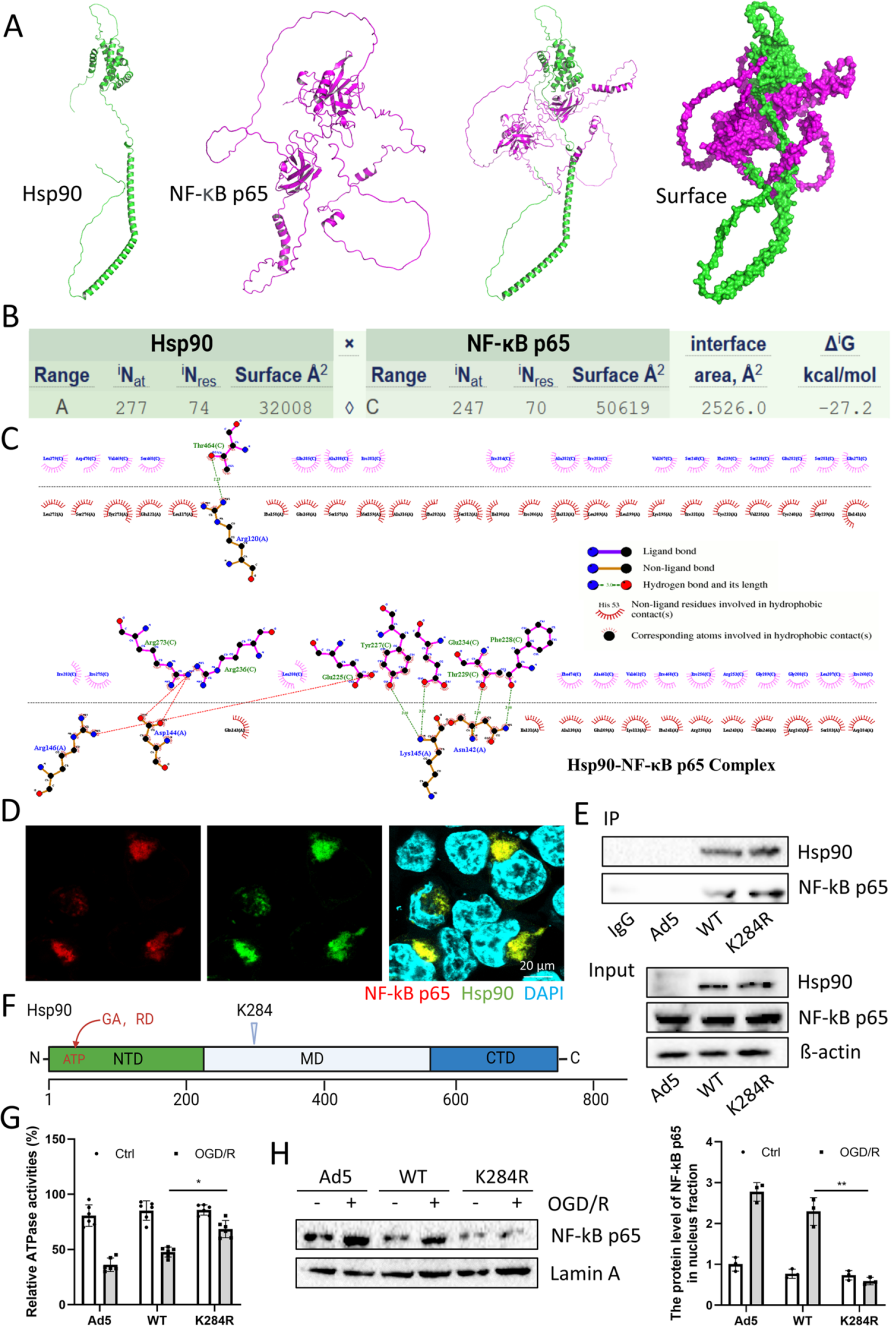
Hsp90 acetylation decreases its interaction with NF-κB p65. (**a**)**.** The protein 3D structures were obtained through the UniProt database: green: Hsp90 (ID: Q7L3B6), purple: NF-kB p65 (ID: Q04206). (**b**)**.** Protein‒protein binding (Hsp90-NF-κB p65) affinity evaluation (kcal/mol). (**c**)**.** Protein–protein 2D interaction diagram of molecular docking. Protein A indicates Hsp90, and protein C indicates NF-kB p65. (**d**)**.** Immunofluorescence staining of the colocalization between Hsp90 and NF-κB p65 under normal conditions by confocal microscopy (magnification: 630x). (**e**)**.** Protein–protein interactions between NF-κB p65 and Hsp90-WT or Hsp90-K284R were detected by Co-IP. (**f**)**.** Domain structure of Hsp90; the K284 site belongs to the MD; GA and RD bind ATP sites in the NTD of Hsp90. (**g**)**.** Detection of ATPase activity after overexpression of Hsp90-WT or Hsp90-K284R. n = 6 for each group. (**h**)**.** The total NF-κB p65 protein expression level in the nucleus was detected by Western blotting. n = 3 for each group. Cardiomyocytes were harvested 1–2 days post OGD/R challenge. Image Lab was used to analyze the protein expression compared with the host-keeping gene Lamin A. *p < 0.05, **p < 0.01, *vs.* OGD/R + Ad5-Hsp90-WT

To clarify the regulatory effect of Hsp90 on inflammatory pathways. We hypothesized that Hsp90 acts as a receptor, docking the NF-κB p65 molecule into its binding pocket, and calculated the fitting of Hsp90-NF-κB p65 using AutoDock Vina 1.1.2 (Fig. 4a). The results showed that the best pose offered calculated binding energy of -27.2 kcal/mol, which intrigued us strongly about the association of Hsp90 with NF-κB p65 (Fig. 4b). Furthermore, the protein 2D interaction diagram of Hsp90-NF-κB p65 revealed that the amino acid 31–331 region of Hsp90 binds to NF-κB p65 (Fig. 4c). This finding further suggested that this region of Hsp90 may be the key to regulating the activation of the NF-kB signaling pathway. Additionally, it is interesting to determine whether K284 acetylation of Hsp90 affects the activation of the NF-κB pathway. From the colocalization results, we found that Hsp90 and NF-κB p65 colocalized in the cytosol (Fig. 4d). Subsequently, Hsp90-WT and Hsp90-K284R were overexpressed in primary cardiomyocytes to check the protein–protein interaction (PPI) with NF-κB p65. Figure 4e showed that Hsp90-WT interacted with NF-κB p65, and the interaction was enhanced after Hsp90-K284R overexpression. According to the previous literature, an important feature of the middle domain (MD) of Hsp90 is that it regulates Hsp90 function by binding the γ-phosphate of ATP specified for the N-terminal domain (NTD), thus modulating its ATPase activities [26]. Since ATPase activities are needed for chaperone cycling and binding of Hsp90 client proteins [27], many natural product inhibitors, such as geldanamycin (GA) [28] and radicicol (RD) [29], have been found to bind Hsp90 by competitively binding ATP sites in the NTD of Hsp90 with ATP (Fig. 4f). Interestingly, K284 is derived from the MD, so whether K284 also regulates ATPase activities has attracted our attention. Surprisingly, the ATPase activity assay results showed that after overexpression of Hsp90-K284R, the ATPase activity was enhanced significantly compared with that in the Hsp90-WT group, but there was no large difference in the Hsp90-WT group compared with that in the Ad5-Vector group (Fig. 4g). This means that the K284 site plays a crucial role in the interaction of Hsp90-NF-κB p65. Furthermore, nuclear protein was extracted after treatment, and the protein level of NF-κB p65 in the nuclear fraction was decreased in the Hsp90-K284R group under OGD/R treatment compared with the Hsp90-WT group (Fig. 4h). In brief, NF-κB p65 was activated after OGD/R treatment in primary cardiomyocytes, and overexpression of Hsp90-K284R could change the ATPase activities of NTD and enhance the interaction between Hsp90 and NF-κB p65, thereby inhibiting the translocation of NF-κB p65 after OGD/R.

### rIL-37 Improved Cardiac Function after I/R in Mice

**Fig. 5:**
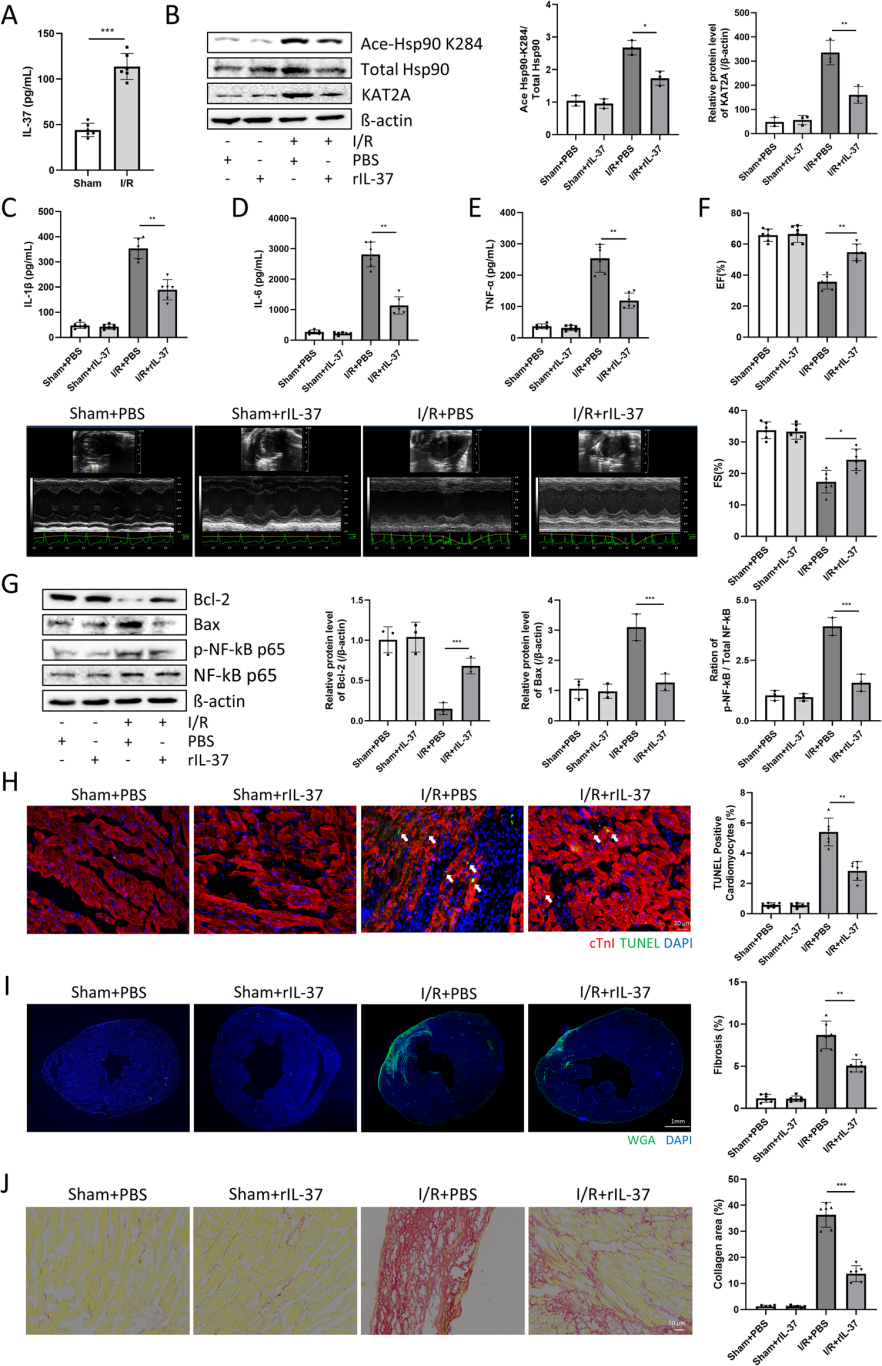
rIL-37 improved cardiac function after I/R in mice. (**a**)**.** Anti-inflammatory cytokines: IL-37 protein levels were detected by ELISA. n = 6 for each group. ***p < 0.001 *vs.* Sham. (**b**)**.** Ace-Hsp90 K284, total Hsp90, and KAT2A protein expression were detected by Western blotting. n = 3 for each group. (**c-e**)**.** Inflammatory cytokines: IL-1β, IL-6, and TNF-α protein levels were detected by ELISA. n = 6 for each group. (**f**)**.** Cardiac function was detected by echocardiography (LVEF% and LVFS%) 28 days post I/R surgery. n = 6 for each group. (**g**)**.** Bcl-2, Bax, phosphorylated NF-κB p65, and total NF-κB p65 protein expression were detected by Western blotting. n = 3 for each group. MI tissues and serum were taken 3 days or 28 days post I/R surgery, respectively. (**h**)**.** Cardiomyocyte apoptosis after 3 days of I/R surgery was detected by TUNEL staining (magnification: 400x; n = 6 for each group). (**i**)**.** Myocardial fibrosis was detected by WGA staining (Magnification: 100x, n = 6 for each group). (**j**)**.** Myocardial fibrosis was detected by picrosirius red staining (magnification: 200x; n = 6 for each group), and MI heart tissues were taken 28 days post I/R surgery. Image Lab was used to analyze the protein expression compared with the host-keeping gene β-actin. ImageJ was used to analyze myocardial fibrosis. *p < 0.05, **p < 0.01, ***p < 0.001 *vs.* I/R + PBS

As the literature shows, IL-37 plays a critical role in inflammation, especially in MI [30]. Initially, our findings (Fig. 5a) affirmed the upregulation of serum IL-37 in MI mice. To further explain the function of IL-37 in I/R and also the relation with Hsp90. The rIL-37 was involved in further experiments. As shown in Fig. 5b, after treatment with rIL-37, the acetylation of Hsp90-K284 was significantly decreased in mice compared with the I/R + PBS group. Notably, Hsp90 K284 serves as the acetylation site for the acetylation enzyme KAT2A (Supplementary Fig. 1) and the protein levels of KAT2A decreased following rIL-37 treatment (Fig. 5b). Additionally, mice in the I/R + rIL-37 group exhibited reduced inflammation levels (Fig. 5c-e, Supplementary Fig. 2b). Echocardiographic results showed that rIL-37 relieved EF% and FS% after I/R in mice (Fig. 5f). In addition, low protein levels of Bcl-2 and high protein levels of Bax and p-p65 in myocardial tissue were reversed after rIL-37 treatment in mice with I/R surgery (Fig. 5g). Moreover, the immunofluorescence staining results showed fewer TUNEL-positive cardiomyocytes and less fibrosis in the rIL-37 treatment group than in the I/R + PBS group (Fig. 5h and i). Picrosirius Red staining also confirmed that myocardial collagen was significantly reduced after rIL-37 treatment (Fig. 5j). However, rIL-37 treatment did not change myofibroblast density after I/R injury (Supplementary Fig. 2d). These results indicated that rIL-37 can affect the acetylation of Hsp90-K284 by regulating KAT2A, significantly decreasing inflammatory levels, and alleviating fibrosis and apoptosis, thereby improving cardiac function.

### rIL-37 Decreased Inflammation and Apoptosis after OGD/R in Primary Cardiomyocytes

**Fig. 6:**
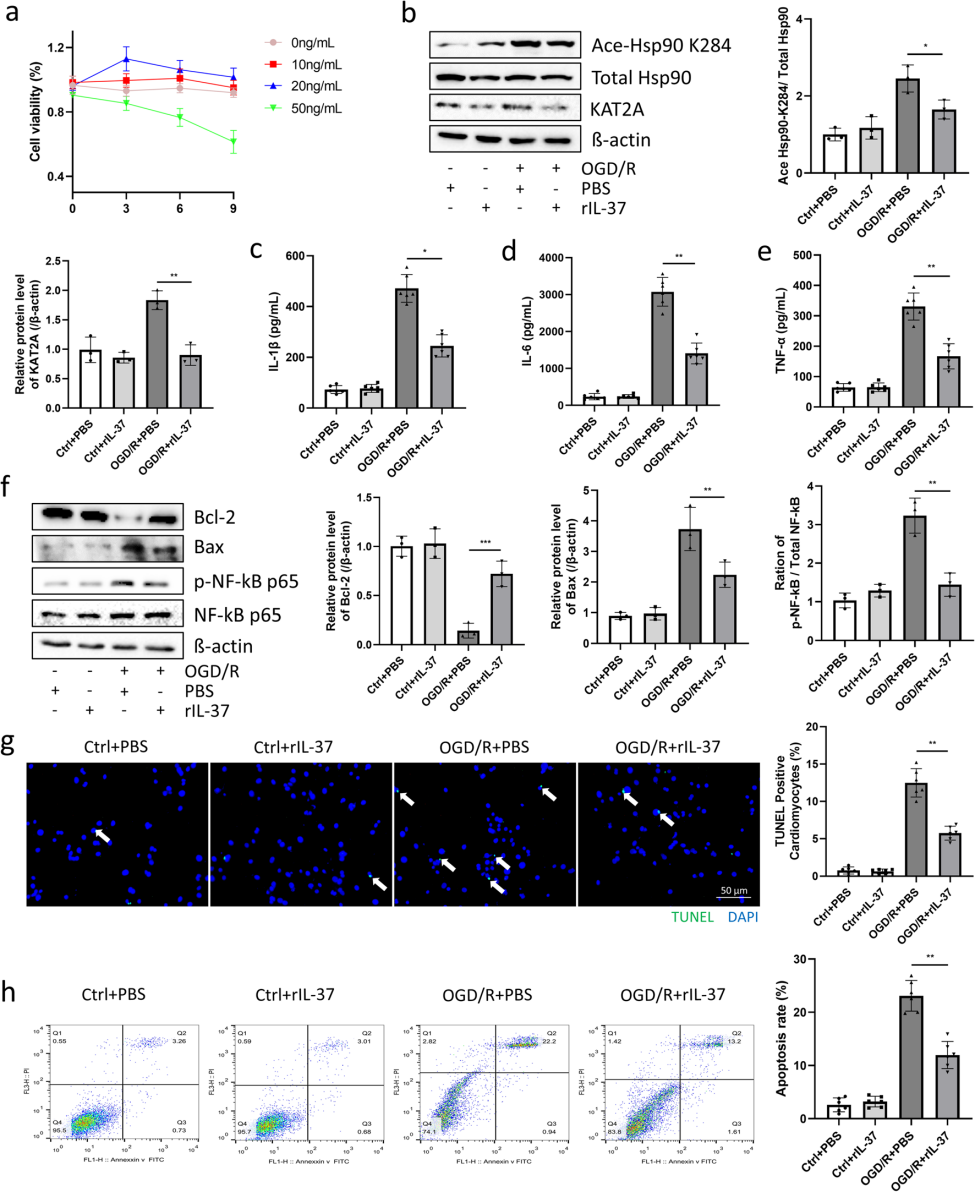
rIL-37 decreased inflammation and apoptosis after OGD/R in primary cardiomyocytes. (**a**)**.** Cell viability of cardiomyocytes after the rIL-37 challenge was detected by CCK8 assay. n = 6 for each group. (**b**)**.** Ace-Hsp90 K284, total Hsp90, and KAT2A protein expression were detected by Western blotting. n = 3 for each group. (**c-e**)**.** Inflammatory cytokines: IL-1β, IL-6, and TNF-α protein levels were detected by ELISA. n = 6 for each group. (**f**)**.** Bcl-2, Bax, phosphorylated NF-κB p65, and total NF-κB p65 protein expression were detected by Western blotting. n = 3 for each group. (**g** and **h**)**.** Cardiomyocyte apoptosis was detected by TUNEL staining and FACS (magnification: 200x; n = 6 for each group). Cardiomyocytes or supernatants were harvested 1–2 days post OGD/R challenge. Image Lab was used to analyze the protein expression compared with the host-keeping gene β-actin. *p < 0.05, **p < 0.01, ***p < 0.001 *vs.* OGD/R + PBS

Next, we detected the effects of rIL-37 in the OGD/R model. First, we examined whether rIL-37 had toxic effects on primary cardiomyocytes. Utilizing a cell viability assay, we determined that the viability of primary cardiomyocytes reached the highest level after incubation with 20 ng/mL rIL-37 for 3 h (Fig. 6a). Moreover, acetylation of Hsp90-K284 in primary cardiomyocytes treated with rIL-37 was significantly lower than that in the OGD/R + PBS group (Fig. 6b). Additionally, the protein levels of inflammatory cytokines were lower in the OGD/R + rIL-37 group than in the OGD/R + PBS group (Fig. 6c-e). Moreover, after the OGD/R challenge, rIL-37 treatment induced higher Bcl-2 expression and lower Bax expression as well as a decreased ratio of p-p65/p65 compared with PBS treatment (Fig. 6f). Compared with the OGD/R + PBS group, rIL-37 treatment reduced the apoptosis of cardiomyocytes induced by OGD/R (Fig. 6g and h). In conclusion, rIL-37 effectively alleviated primary cardiomyocyte apoptosis and inflammation induced by OGD/R.

## Discussion

In this study, we discovered that Hsp90 acetylation in cardiomyocytes is induced by I/RI. Additionally, the presence of acetyl-Hsp90 K284 hindered the formation of the Hsp90-NF-κB p65 complex, leading to the translocation of NF-κB p65 to the nucleus. Subsequently, we identified rIL-37, as a potential inhibitor against KAT2A, illustrating its capacity to promote cardiac protection during I/RI. This investigation not only sheds light on the role of rIL-37 in I/RI but also provides compelling evidence for the potential clinical applications of rIL-37 in the treatment of I/RI.

Numerous studies have reported the significant roles of protein acetylation in the pathogenesis of cardiovascular diseases (CVDs) [31,32]. The mitochondrial deacetylase Sirt3 has been implicated in the amelioration of vascular dysfunction and hypertension by attenuating vascular inflammation and oxidative stress [33]. Our findings aligned with these studies, demonstrating that reduced acetylation of Hsp90-K284 contributes to the mitigation of ischemia-induced heart injury. Furthermore, previous research has indicated the involvement of histone acetylation in the hearts of MI mice [34]. Contradictory findings suggest a potential association between cardiomyocyte cell cycle activation and histone acetylation following MI [35]. In addition, Wang et al*.* reported that N4-acetylcysteine is involved in the reduction of cardiomyocyte apoptosis^9^. In addition to the acetylation of Hsp90, it is possible that histone acetyltransferase might acetylate other proteins, and at the same time, histone deacetylases (HDACs) might also deacetylate these proteins. However, different classes of HDACs have different functions in the heart. For example, class II and class III HDACs have a protective role [36,37], whereas class I HDACs have deleterious effects on the heart [38]. It is still a long way for us to develop a good understanding of the molecular mechanisms of protein acetylation and deacetylation.

The Hsp90 monomer is composed of three essential structural domains that are pivotal for its activity: the N-terminal domain (NTD), middle domain (MD), and C-terminal domain (CTD). The NTD is primarily responsible for ATPase activity, which drives the conformational cycle of the enzyme [39,40]. On the other hand, the CTD is implicated in the dimerization of Hsp90 monomers, leading to the formation of functional Hsp90 enzymes, and plays a critical role in facilitating interactions with cooperative partners [41]. The MD interacts with substrate (or client) proteins, serves as binding sites for cochaperones, and participates in ATP hydrolysis [26,42]. According to the results of molecular docking, colocalization, and immunoprecipitation, Hsp90 and NF-κB p65 combine in the cytoplasm, and the mutation of K284, which is derived from MD, can further stabilize the physical interaction between the two proteins. Furthermore, during OGD/R treatment, the ATPase activity of cardiomyocytes was significantly compromised, and the overexpression of Hsp90 failed to reverse this phenotype. Intriguingly, the introduction of Hsp90-K284R led to a substantial restoration of ATPase activity, suggesting the potential of Hsp90 to regain its functional efficacy under these conditions.

Considering that the ATPase binding site is necessary for chaperone cycling and binding to Hsp90 client proteins, we explored the role of Hsp90 (K284) in inflammatory signaling pathways. Inflammation is a vital bodily response of the body to MI [43]. It helps tissue repair and healing, yet persistent inflammation can lead to detrimental harmful heart remodeling [44]. The NF-κB gene serves as a key regulator of inflammation and immune homeostasis, playing a crucial role in inflammatory diseases such as MI [45]. Activation of NF-κB signaling leads to the increased expression of downstream proinflammatory cytokines, including IL-1β, IL-6, and TNF-α, contributing to apoptosis, fibrosis and cardiac dysfunction [46]. Furthermore, Liang Ge has suggested that IL-1β can be secreted through secretory autophagy, with this process being closely associated with Hsp90 [47]. Our research demonstrated elevated levels of IL-1β, IL-6, and TNF-α in the plasma of I/R mice. However, following the overexpression of Hsp90-K284R, a notable amelioration of the inflammatory response was observed. This observation suggested that the K284R mutation can indeed impact the regulatory role of Hsp90 in the NF-KB signaling pathway.

Additionally, we isolated nuclear protein from cardiomyocytes transfected with Ad5-Hsp90-WT and Ad5-Hsp90-K284R, with or without OGD/R challenge. The results indicated a reduction in the nuclear fraction of the NF-κB p65 protein in the Ad5-Hsp90-K284R group following OGD/R challenge. This finding further confirmed that the K284R mutation hinders the nuclear translocation of NF-κB p65. Meng et al*.* previously reported that Hsp90 offers cardioprotection in ischemic postconditioning by suppressing inflammation, apoptosis, and myocardial injury [48]. Hsp90 was also reported to reduce NF-κB signaling pathway activation [49]. Our findings revealed that the acetylation of Hsp90 at K284 introduces a novel mechanism for modulating the activity of the NF-κB signaling pathway. Therefore, our data demonstrated that acetyl-Hsp90 K284 impacts the complex formation between Hsp90 and NF-κB p65, leading to increased NF-κB p65 phosphorylation and nuclear translocation. However, the overexpression of Hsp90 did not yield a significant effect on NF-κB p65 activity.

The imbalance of inflammatory response and cytokine expression plays a pivotal role in myocardial ischemia. Therefore, how to regulate the inflammatory balance after MI has always been a hot topic in research. Interestingly, the role of IL-37 in the IL-1 family in regulating inflammation has been discovered by researchers [50]. Upregulated IL-37 can inhibit proinflammatory cytokines, such as IL-1β, IL-6 [51]. A more interesting thing is that IL-37 can regulate the activation of NF-κB signaling pathway [52,53]. Nevertheless, the precise mechanisms of underlying the improvement of cardiac function by IL-37 after I/RI remain obscure. In this study, we identified that rIL-37 could regulate the expression of KAT2A, thereby inhibiting the acetylation of Hsp90, and consolidating the binding of Hsp90 and NF-κB in the cytoplasm. Through our in vivo and in vitro experiments, we observed that rIL-37 effectively improved cardiac function following I/R or OGD/R challenges without exhibiting any toxic effects on mice or cells. As in the study by Zeng et al*.,* they administered rIL-37 intervention to mice before and after MI surgery, and found that rIL-37 significantly improved the cardiac function of mice one day, seven days, and 28 days after MI, including cardiomyocyte apoptosis, myocardial fibrosis and inflammatory response^20^. Therefore, we believe that early administration of rIL-37 has a protective effect on MI intervention. Mechanically, our results indicated that the cardioprotective effect of rIL-37 is achieved by reducing the acetylation of Hsp90 at the K284 sit via KAT2A.

In conclusion, our study demonstrated the enhancement of acetylated Hsp90 after I/R in vivo or induction by OGD/R in vitro. Acetyl-Hsp90 K284 impacted the complex formation of Hsp90 with NF-κB p65, resulting in the activation of the NF-κB signaling pathway, thereby inducing inflammation and apoptosis. Site-directed mutagenesis of K284 in Hsp90 mitigated inflammation, apoptosis, and fibrosis and led to an improvement in cardiac function. Consequently, the regulation of acetyl-Hsp90 in cardiomyocytes may present a novel therapeutic strategy for MI in the future. What’s more, rIL-37 inhibited the expression of KAT2A, thereby reducing the acetylation level of Hsp90-K284. This property renders it a potential candidate for the development of drugs aimed at improving cardiac function after I/RI.

## Disclosures

No human studies were carried out by the authors for this article.

All animal experiments were performed in strict accordance with the Guidelines for the Care and Use of Laboratory Animals (NIH Publication, revised 2011), which were also approved by the Animal Ethical Care Committee of the Third Affiliate Hospital of Qiqihar Medical University (AECC-2024–002).

## Supplementary Information

Below is the link to the electronic supplementary material.

Supplementary file1 (TIF 341 KB)

Supplementary file2 (TIF 12176 KB)

## Data Availability

All data that support the findings in this study are available from the corresponding author upon reasonable request.

## Abbreviations

(CVDs): Cardiovascular diseases
(I/RI): Ischemia‒reperfusion injury
(AMI): Acute myocardial infarction
(Hsp90): Heat shock protein 90
(HDAC): Histone deacetylase
(rIL-37): Recombinant interleukin-37
(GA): Geldanamycin
(RD): Radicicol
(NTD): N-terminal domain
(MD): Middle domain
(NTD): C-terminal domain
(Ad): Adenovirus
(rAAV): Recombinant adeno-associated virus
(CMV): Cytomegalovirus
(KAT): Lysine acetyltransferase
(KDAC): Lysine deacetylase
(LVEDD): Left ventricular end-diastolic diameter
(LVESD): Left ventricular end-systolic diameter
(FS%): Fractional shortening
(EF%): Ejection fraction
(PBS): Phosphate-buffered saline
(PFA): Paraformaldehyde
(OCT): Optimal cutting temperature
(DMEM): Dulbecco's Modified Eagle's Medium
(FBS): Fetal bovine serum
(OGD): Oxygen–glucose deprivation
(WGA): Wheat Germ Agglutinin
(α-SMA): Alpha smooth muscle actin
