# Supplementary figures and images for: Inhibition of Hsp90 K284 Acetylation Aalleviates Cardiac Injury After Ischemia–Reperfusion Injury
Source: J Cardiovasc Transl Res. 2024 Jul 24;17(6):1427–41. doi: 10.1007/s12265-024-10548-0 (PMC11634933; doi:10.1007/s12265-024-10548-0)

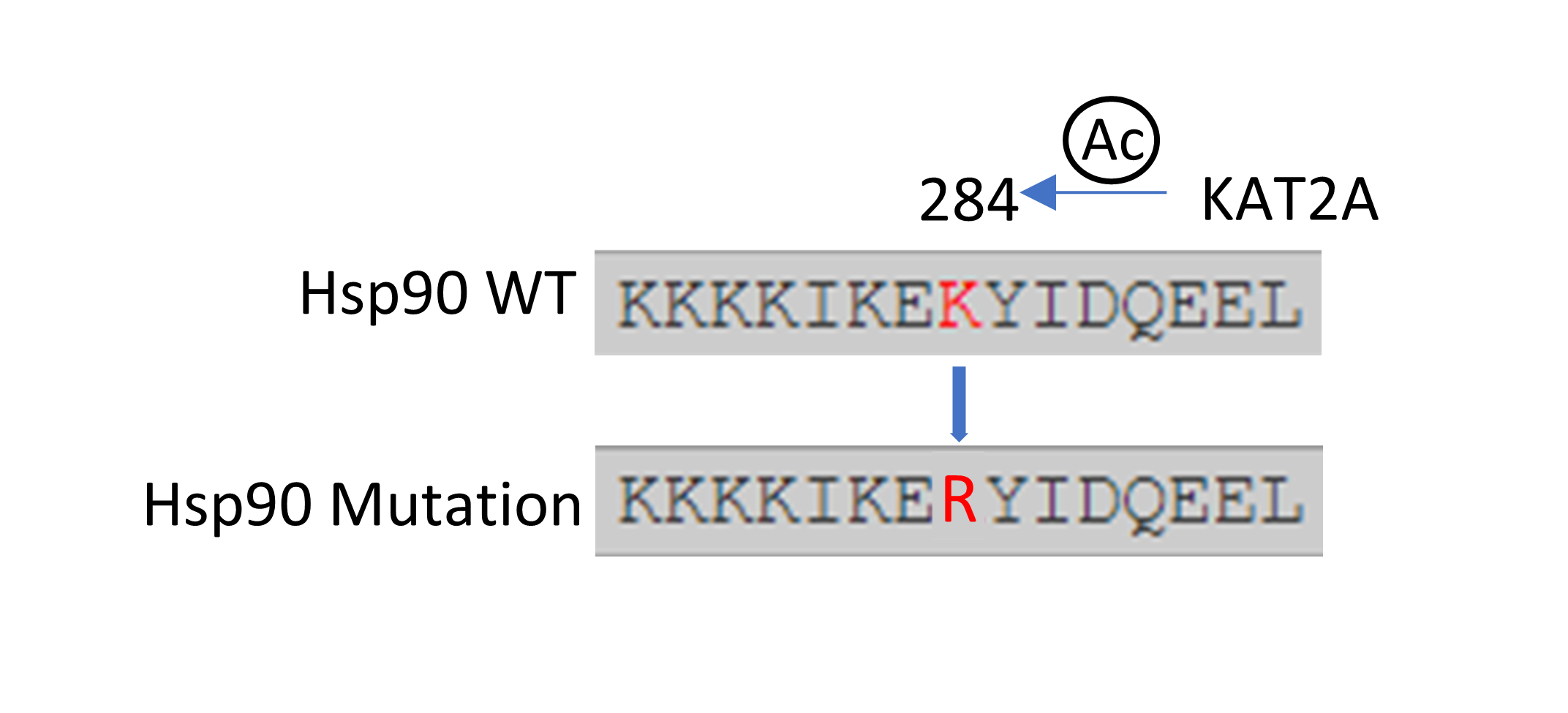

Supplement: Supplementary file 1 — Supplementary file1 (TIF 341 KB) [file 12265_2024_10548_MOESM1_ESM.tif]

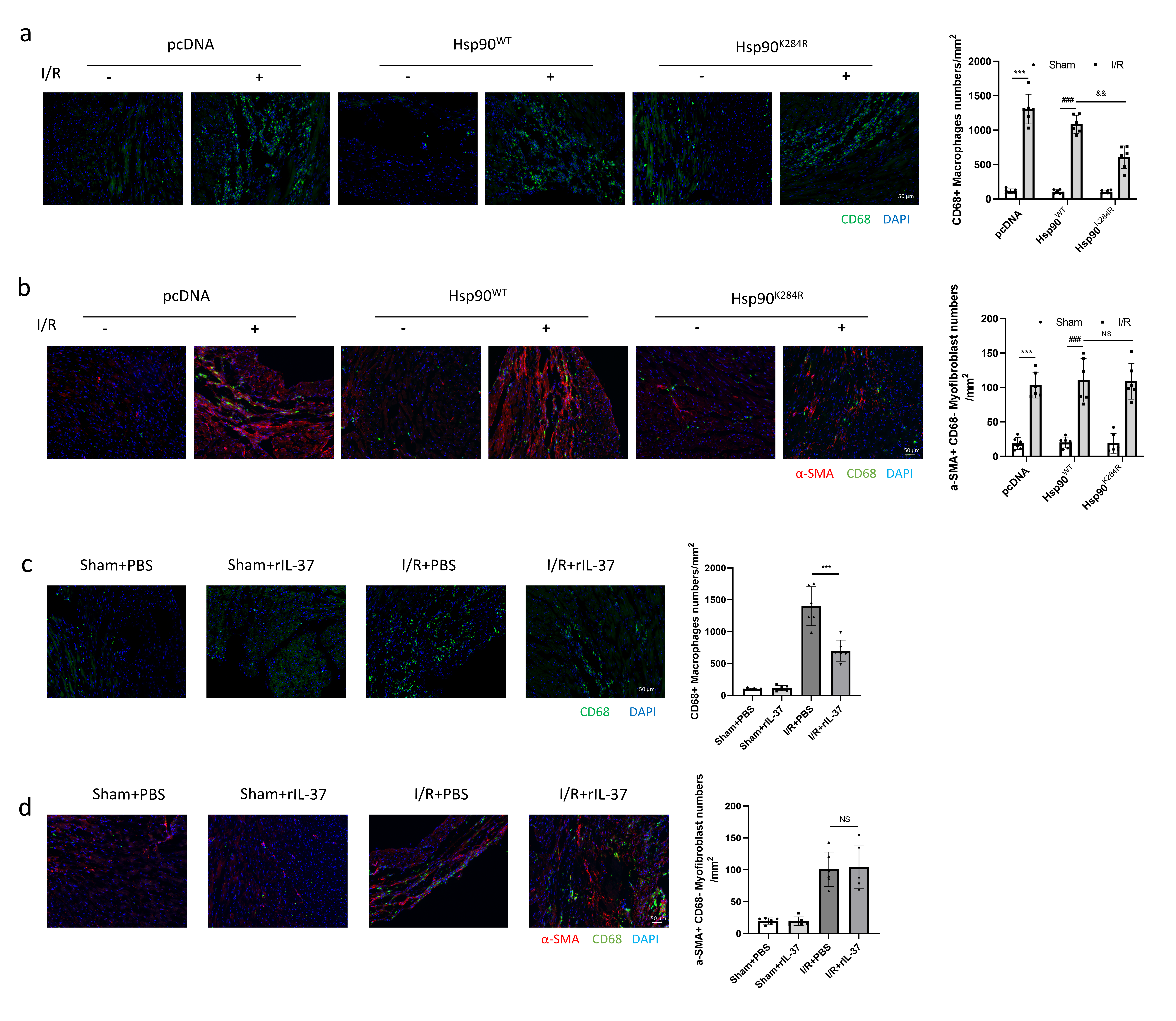

Supplement: Supplementary file 2 — Supplementary file2 (TIF 12176 KB) [file 12265_2024_10548_MOESM2_ESM.tif]
